# Direct and indirect costs associated with ankylosing spondylitis and related disease activity scores in Turkey

**DOI:** 10.1007/s00296-015-3236-y

**Published:** 2015-03-07

**Authors:** Nurullah Akkoç, Haner Direskeneli, Hakan Erdem, Ahmet Gül, Yasemin Kabasakal, Sedat Kiraz, Dilara Balkan Tezer, Başak Hacıbedel, Vedat Hamuryudan

**Affiliations:** 1 Division of Rheumatology and Immunulogy, Department of Internal Medicine, Faculty of Medicine, Dokuz Eylül University, Kültür Mh., Cumhuriyet Blv No: 144, 35210 Konak, Izmir Turkey; 2 Department of Rheumatology, Pendik Training and Research Hospital, Marmara University, Istanbul, Turkey; 3 Department of Infectious Diseases and Clinical Microbiology, Gülhane Military Medical Academy, Ankara, Turkey; 4 Division of Rheumatology, Department of Internal Medicine, Istanbul Faculty of Medicine, Istanbul University, Istanbul, Turkey; 5 Division of Rheumatology, Department of Internal Medicine, Faculty of Medicine, Ege University, Izmir, Turkey; 6 Division of Rheumatology, Department of Internal Medicine, Faculty of Medicine, Hacettepe University, Ankara, Turkey; 7 Pfizer Pharmaceuticals, Istanbul, Turkey; 8 Division of Rheumatology, Depertmant of Internal Medicine, Cerrahpaşa Faculty of Medicine, Istanbul University, Istanbul, Turkey

**Keywords:** Ankylosing spondylitis, Direct costs, Indirect costs, Turkey

## Abstract

This study assessed quality of life, direct and indirect healthcare costs related to ankylosing spondylitis (AS). This study included 650 prevalent AS patients visiting seven centers at tertiary healthcare institutions in Turkey who were interviewed using a standard questionnaire to determine annual direct and indirect healthcare costs. Eligible patients were age ≥18 years with AS for at least 12 months. Direct costs were categorized as inpatient, outpatient and pharmacy, and AS-related consultation. Indirect costs were categorized as workday loss, additional AS-related costs, and caregiver costs. Clinical outcome measures were obtained, including Patients’ Global Disease Activity (Pt-GDA); visual analog scale (Pain-VAS) for pain; Bath Ankylosing Spondylitis Disease Activity Index (BASDAI), Functional Index (BASFI), and Metrology Index (BASMI) scores, and EuroQoL 5 dimension (EQ-5D) health status survey scores. Mean (€4,335.20) and median (€5,671.00) annual costs per patient were calculated. Pharmacy costs (€4,032.73) were highest among overall expenditures, followed by additional AS-related consultation (€2,480.38), outpatient (€225.02), and inpatient costs (€29.98). Over half of AS patients (54.8 %) experienced work loss. Related average annual costs were €414.16, based on income level. 10.3 % of AS patients incurred an additional €2,008.07 in 1 year. 6.8 % of patients required caregivers and incurred €778.70 in average annual patient paid costs. Mean Pt-GDA, Pain-VAS, EQ-5D, BASDAI, BASFI, and BASMI scores were 4.4, 40.5, 62.7, 3.6, 3.1, and 2.9, respectively. Direct and indirect AS-related costs are high and represent a considerable economic burden on Turkish AS patients.

## Introduction

Ankylosing spondylitis (AS) is a chronic, systematic, and inflammatory rheumatic disease that primarily affects the axial skeleton and can lead to structural and functional impairments, low productivity, decrease in life quality, and substantial healthcare resource use [1]. Clinical features of AS include back pain, stiffness, asymmetrical peripheral oligoarthritis, and specific organ involvement such as anterior uveitis, psoriasis, and chronic inflammatory bowel disease [2].

Approximately, 80 % of patients develop their first symptoms before age 30 and less than 5 % develop AS when they are over age 45. Men are more likely to be affected compared to women at an approximate 2:1 ratio [3]. Also, juvenile-onset AS is usually associated with worse outcomes than adult-onset AS [4]. Reported AS prevalence ranges between 0.1 and 0.8 %, and recent studies suggest that prevalence is actually closer to 0.5 % in European populations, especially in Northern Europe [5]. Surveys have estimated AS prevalence in the Turkish adult population at 0.49 % [6].

Traditionally, the “gold standard” of AS treatment is non-steroidal anti-inflammatory drugs (NSAIDs), which improve inflammatory symptoms of AS including the inflammatory back pain. Furthermore, gastrointestinal complications are a common side effect of NSAIDs [7]. Disease-modifying antirheumatic drugs (DMARDs), including sulfasalazine, methotrexate, and leflunomide, though not proven effective, are widely used in some countries as a secondary treatment approach for patients who are refractory to NSAID treatment.

In recent years, anti-tumor necrosis factor alpha (TNFα) use has been studied extensively and has been shown to provide significant clinical benefits for AS patients. Available TNFα inhibitors are infliximab, adalimumab, etanercept, golimumab, and certolizumab. These drugs strongly suppress inflammation [8].

Prior to development of efficacious biologics, overall treatment costs were low in AS patients. Annual estimates included €2,335 in the Netherlands, €2,064 in France, €1,572 in Belgium, and €1,750 in the United States [7, 9]. However, availability of improved but expensive treatment options required the need for information concerning the current disease burden. Many recent studies have been implemented around the world to estimate the economic burden of AS per patient in different countries. In the United Kingdom, total annual direct costs per patient were calculated at £15,973 [10]. In Spain, the total mean annual cost per patient was estimated at €20,328, with direct costs accounting for 22.8 % [11]. In Canada, the mean annual cost per patient was $9,008 Canadian dollars (€7,383), of which direct healthcare costs represented 62.0 %, or $5,585 (€4,578) [12]. In Hong Kong, total annual costs were calculated at €7,045, of which direct costs accounted for 38.0 % [13].

The economic burden of AS in Turkey is not well documented. This study aimed to examine AS patient characteristics and the economic impact of the disease. In this respect, the study objectives were to evaluate AS patient demographic and clinical characteristics, examine patient work environment and prescribed medication types, estimate direct and indirect costs of AS, and determine the association between cost and disease activity score. The present study also aimed to highlight the significance of the economic burden of AS inform policy decision makers to better accommodate Turkish employees diagnosed with AS.

## Methods

In the present noninterventional, cross-sectional study, information was collected directly from patients through a questionnaire distributed to seven university hospitals, tertiary healthcare centers in Turkey. All subjects were at least age 18 years, with an AS diagnosis for a minimum of 12 months. Patients were interviewed in a single visit during the 1-year study period from May 2011 through August 2012. Patients were interviewed regarding demographic information (age, gender, and region), AS-related healthcare services including rheumatologic care, prescription medications, rehabilitative therapies, diagnostic and therapeutic procedures (including complementary therapies), inpatient hospitalization, and medications used during hospitalization, comorbid conditions (heart disease, diabetes), medication use (DMARDs, non-selective Cox inhibitors) during the 3-month pre-index period, as well as expenditures for medical devices, caregiver needs, and work capacity (sick leave days).

Clinical outcome measures were collected for Patients’ Global Disease Activity (Pt-GDA) assessment, EuroQoL 5 dimension (EQ-5D) health status survey, pain score on VAS, Bath Ankylosing Spondylitis Disease Activity Index (BASDAI) [14], Bath Ankylosing Spondylitis Functional Index (BASFI) [15], and the Bath Ankylosing Spondylitis Metrology Index (BASMI) [16]. Pt-GDA, BASDAI, BASFI, and BASMI results are measured on a scale of 0–10, where higher scores indicate higher disease activity. EQ-5D and pain scores were measured on a VAS of 0–100 mm. High pain VAS, BASDAI, and BASFI scores indicate higher pain severity, and higher EQ-5D scores indicate better quality of life.

Total direct medical costs were calculated as the summation of inpatient, outpatient, pharmacy costs, and other AS-related interventions (e.g., acupuncture, homeopathic, other). Total indirect costs were calculated as the sum of those due to work loss, caregiver, and other expenses such as new car, apartment, or special equipment purchases due to AS. Annual costs for outpatient care and pharmacy, work loss, caregiver, and any consultation expenses that are not covered by social insurance were calculated by multiplying 3-month data by four, and data for additional AS-related investments were collected for 1-year period. For work loss, income ranges were taken into account, and the human capital approach was used. The direct and indirect costs were expressed per year. All costs were expressed in 2011 euros (€1.00 = 2.3 Turkish Liras, without inflation adjustment).

Univariate statistics for demographic and clinical variables were created for descriptive analysis. Continuous variables were summarized by providing the number of observations, mean and standard deviation (SD), and median (minimum–maximum). Numbers and percentages are provided for dichotomous and polychotomous variables. Chi-square test was used to compare categorical variables. All statistical analyzes were conducted using SAS v.9.3 and STATA v11 software.

## Results

After applying all inclusion criteria, the analytic sample included 648 patients. Mean patient age was 40.5 years, and 98.6 % of them were under age 65 years. Almost one-third of the patients were women. Average disease duration was 7.7 years. Nearly, 8 % of the study subjects were diagnosed with at least one of the following comorbid conditions: heart disease, diabetes, respiratory disease, allergies, Crohn’s disease, uveitis, or rheumatoid arthritis. Of the patients, 7.5 % were hospitalized, 3.5 % were in rehabilitation services, 2.1 % underwent surgery, and 1.2 % had prosthesis (Table 1).

**Table 1:** Demographic and clinical characteristics of the study participants

All patients	*n* = 648
Age, mean (SD)	40.6 (11.4)
≤65 years, *n* (%)	639 (98.6)
>65 years, *n* (%)	9 (1.4)
Sex (female), *n* (%)	231 (35.7)
Pre-index comorbid conditions, *n* (%)	
Uveitis	49 (7.6)
Heart disease	33 (5.1)
Diabetes	31 (4.8)
Allergy	26 (4.1)
Respiratory disease	22 (3.4)
Crohn’s disease	21 (3.2)
Rheumatoid arthritis	13 (2.1)
Disease duration, years, mean (SD)	7.7 (7.4)
Symptom duration, years, mean (SD)	5.7 (6.3)

*SD* standard deviation, *DMARDs* disease-modifying antirheumatic drugs

Interestingly, the percentage of female patients who were prescribed biologic agents was lower than for male patients. In addition, average Pt-GDA, Pain-VAS, BASDAI, and BASFI scores were found to be significantly lower in males than in females, whereas EQ-5D and BASMI scores were similar (Table 2).

**Table 2:** Clinical variables related to disease burden

	Entire population (*n* = 648)	Female (*n* = 231)	Male (*n* = 417)	*p* value
Pt-GDA	4.4 (2.2)	4.7 (2.1)	4.2 (2.2)	0.0039
Pain-VAS	40.5 (24.1)	45.7 (23.2)	37.6 (24.1)	<0.0001
BASDAI	3.6 (2.2)	4.2 (2.2)	3.2 (2.1)	<0.0001
BASFI	3.1 (2.5)	3.5 (2.4)	2.9 (2.5)	0.0034
BASMI	2.9 (2.6)	2.6 (2.3)	3.1 (2.8)	0.0610
EQ-5D	0.63 (20.5)	0.61 (19.6)	0.64 (20.9)	0.0710

Data are presented as mean (SD)

*Pt*-*GDA* global disease activity, *VAS* visual analog scale, *BASDAI* bath ankylosing spondylitis disease activity index, *EQ*-5*D* euroQol health status, *BASFI* bath ankylosing spondylitis functional index, *BASMI* bath ankylosing spondylitis metrology index

Most patients were prescribed biologic agents, DMARDs, and NSAIDs (66.5, 50.5, and 37.7 %), followed by gastrointestinal-related medications such as proton pump inhibitors, histamine h2-receptors (28.9 %), and glucocorticoids (10.34 %) (Table 3).

**Table 3:** Medication use for ankylosing spondylitis patients during the 3-month pre-index period

	All patients, (*n* = 648) *n* (%)
Biologic agents	431 (66.5)
DMARDs	327 (50.5)
NSAI agents	244 (37.7)
NSAI topical agents	131 (20.2)
Paracetamol	17 (2.6)
Glucocorticoids	67 (10.3)
Gastroprotective agents	187 (28.9)

*DMARD* disease-modifying antirheumatic drug, *NSAI* non-steroidal anti-inflammatory

Table 4 shows the direct and indirect costs related with AS. Average annual healthcare costs for AS patients were calculated at €4,335.20. The most significant share of overall expenditures consisted of pharmacy costs (€4,032.73), followed by AS-related consultations (€2,480.38), outpatient (€225.02), and inpatient costs (€29.98) (Table 4). A minority of patients (2 %) had other AS-related therapies not covered by social insurance (acupuncture, homeopathy, other), bringing their average annual burden to €2,480.38. Indirect AS-related costs due to work loss, additional AS-related costs, and caregiver costs were evaluated (Table 4). Nearly, 55 % of AS patients were employed, of which 59.4 % had sick leave with the permission of the employer, costing an average of €414.16 annually due to workday loss. Nearly, one-tenth of AS patients incurred additional AS-related costs (e.g., need for new car, apartment, special equipment) at €2,008.07 in 1 year. The utilization of health caregivers and the average annual out-of-pocket cost for caregivers was €778.70 (Table 4).

**Table 4:** Annual average direct and indirect costs of ankylosing spondylitis

	*n*	Annual average costs (SD)	Median (IQR, 25th–75th percentile)
Direct costs			
Inpatient		€29.98 (€119.51)	€0.00 (0.0–8.0)
Outpatient		€225.02 (€190.28)	€166.33 (92.0–245.0)
Pharmacy		€4,032.73 (€2,723.55)	€5,426.56 (1204.0–6225.0)
AS-related consultations		€2,480.38 (3,479.18)	539 (209.0–3043.0)
Total	648	€4,335.20 (€2.891,74)	€5,671.00 (1370.0–6451.0)
Indirect costs			
Additional AS-related costs	67	€2,008.07 (7,376.04)	87 (20.0–435.0)
Caregiver	44	€778.70 (1,179.01)	522 (278.0–870.0)
Workday loss	355	€414.16 (1,313.35)	104 (0.0–319.0)

*AS* ankylosing spondylitis, *SD* standard deviation, *IQR* interquartile range

## Discussion

A variety of studies in the United States and Europe exist evaluating the economic burden and prevalence of AS. Epidemiologic studies have indicated that AS is more prevalent than previously thought [17]. Prevalence of AS as well as its economic burden have been studied only in a limited number of studies in Turkey [6, 18, 19]. Thus, the current study is designed to thoroughly examine AS patient characteristics and the economic impact of the disease in Turkey.

The most significant strength of this study is that it was performed using real-world data. No previously published studies include such data. A recent study by Malhan et al. [20] examined direct and indirect costs of AS patients in Turkey, which was limited because the data were gathered from an expert panel. Although an expert panel is a relatively inexpensive and rapid tool to produce a synthetic judgment based on qualitative and quantitative data, it has some limitations. Comparing opinions often leads to under evaluation of lesser known or underreported points of view. It represents the consensus of panel attendants rather than real-world outcomes [21, 22]. Total direct medical costs per patient differ significantly between the study by Malhan et al. [20] and this study (€3,566 and €4,335.20, respectively).

In addition to direct medical costs, AS patients in Turkey experienced work productivity loss, contributing to the total economic burden. Average annual workday losses in our study (€414.16) exceeded the estimate by Malhan et al. [20] (€245.50). According to Boonen et al. [23], patients in France (*n* = 53) had adjusted productivity losses (using the friction method) totaling €428 per year, and patients in Belgium (*n* = 26) and the Netherlands (*n* = 130) experienced adjusted productivity losses of €476 and €1,257, respectively. Adjusted work disability was 23 % in France, 9 % in Belgium, and 41 % in the Netherlands. In a more recent study conducted by Kvamme et al. [24], annual adjusted productivity loss, using the friction method, for hospitalized patients in the Netherlands reached €7,686 for synthetic DMARD use (*n* = 49) and €8,186 for biologic DMARD use (*n* = 137). The variation in work status and productivity costs observed in European countries suggests that economic generalizability may be limited. This underscores the importance of conducting health economic studies specific to the country of interest for the effective allocation of resources and other healthcare applications. Other indirect cost measures in our research included AS-related complementary therapies and additional costs, as well as out-of-pocket costs for caregiver utilization. These variables were not assessed in the previous studies from Turkey.

The present study is unique in that it uses real-world data, assesses variables not previously examined in other studies and includes a thorough analysis of nationally representative direct and indirect costs of AS in Turkey. It should be noted that all patients were diagnosed at tertiary medical centers, which may explain the unexpectedly high proportion of AS patients receiving biologic therapy, which may be considered as a limitation of the study. It should be noted that use of biologic therapy was less prevalent among female patients than among male patients. That is most probably due to the relatively common presence of fibromyalgia accompanying AS in female patients, which negatively affects disease activity and functional scores as previously reported [25]. It might have been also due to differences in socioeconomic status. It has been shown that groups with lower socioeconomic status seem to have less access to biologic medications, in terms of lower availability, affordability, and acceptability. More barriers that may have resulted in worse disease course in female patients [26]. More hospitalized patients were administered infliximab, possibly because the medication was dispensed via percutaneous infusion, which is a possible weakness of the present study.

Our results also indicated higher disease activity scores among female patients compared to males. Previous studies have also shown that there are gender differences regarding autoimmune disease characteristics, with women scoring higher on pain and quality of life measurement scales [27, 28]. However, these scores were considered higher subjectively and not objectively, suggesting that disease activity may have been discounted in the treatment decision.

AS has significant economic implications for individuals and society. Yet for Turkey, the cost of care has not been analyzed using real-world clinical data. This study also demonstrated that indirect AS-related costs in Turkey are significant. The young age of patients who reported sick leave reveals the negative effect of AS on national economy. For this reason, indirect cost should be taken into consideration during Payor’s decision making process. Moreover, future comparative effectiveness studies concerning AS treatment should include direct and indirect costs.
